# New tools to uncover old tricks: an update on the knowledge on the most successful invasive freshwater helminth, *Schyzocotyle acheilognathi*

**DOI:** 10.1007/s00436-025-08585-y

**Published:** 2025-12-08

**Authors:** Didier Casanova-Hernández, Carlos Daniel Pinacho-Pinacho, Miguel Calixto-Rojas, Miguel Rubio-Godoy, Essicka A. García-Saldaña, Ernesto Velázquez-Velázquez, Jesús Alejandro Zamora-Briseño

**Affiliations:** 1 Programa de Doctorado en Ciencias en Biodiversidad y Conservación de Ecosistemas Tropicales, Instituto de Ciencias Biológicas, Universidad de Ciencias y Artes de Chiapas, Libramiento Norte Poniente 1150, Tuxtla Gutiérrez, Chiapas 29039 México; 2 Investigador Por México, Secihti, Instituto de Ecología A.C., Red de Estudios Moleculares Avanzados, Carretera Antigua a Coatepec 351, El Haya, Xalapa, Veracruz 91073 México; 3 Red de Estudios Moleculares Avanzados, Instituto de Ecología A. C., Carretera Antigua a Coatepec 351, El Haya, Xalapa, Veracruz 91073 México; 4 Red de Biología Evolutiva, Instituto de Ecología A. C., Carretera Antigua a Coatepec 351, El Haya, Xalapa, Veracruz 91073 México; 5 Museo de Zoología, Instituto de Ciencias Biológicas, Universidad de Ciencias y Artes de Chiapas, Libramiento Norte Poniente No. 1150, Tuxtla Gutiérrez, Chiapas 29039 México

**Keywords:** Generalist parasite, Copepod intermediate hosts, Freshwater fish, Omics, Ecological niche modeling, Asian fish tapeworm

## Abstract

The Asian fish tapeworm (AFT), *Schyzocotyle acheilognathi*, is a highly invasive and pathogenic parasite of freshwater fishes, present on all continents except Antarctica. Globally, 310 + definitive fish host species have been recorded, with Mexico showing the highest number of reports. Here, we summarize the current knowledge about the biology, distribution, and ecological determinants of this parasite, highlighting key knowledge gaps that could guide future research. Considering Mexico has quite comprehensive records of this parasite (both in host and geographical terms), we propose that this country can be considered as a large-scale ecological laboratory to investigate the environmental variables associated with the parasite’s dispersion capabilities in different geographic areas, potentially providing clues on the (unexplored) paths followed by this extremely successful invasive parasite. Using published parasitological data, we implemented exploratory analyses of ecological and environmental parameters to identify factors associated with the occurrence of *S. acheilognathi*. Temperature, precipitation, and elevation emerged as potential drivers of its distribution, providing a basis for ecological niche modeling and for predicting future range expansions under climate change scenarios. Despite its extensive host range, *S. acheilognathi* exhibits low genetic diversity—an intriguing feature that may underlie its ecological plasticity and invasive success. We also discuss the growing potential of omics and environmental DNA tools to advance non-destructive detection, clarify host-parasite dynamics, and uncover molecular mechanisms underlying host adaptation and invasion. Integrating these ecological and genomic perspectives will be essential to understand the evolutionary ecology of *S. acheilognathi* and to anticipate its impacts on freshwater biodiversity in an era of global change.

## Introduction

The Asian fish tapeworm (AFT), *Schyzocotyle acheilognathi* (Yamaguti 1934), formerly known as *Bothriocephalus acheilognathi* (WoRMS 2025), is considered the most successful invasive parasite of freshwater fishes (Kuchta et al. 2018). Described in 1934 from a Japanese cyprinid, *Acheilognathus rhombeus*, *S. acheilognathi* was only reported in the Orient (Japan, China, and Russia’s—then the USSR’s—Far East) during the first half of the XX^th^ century. The second half of the XX^th^ century saw its relatively rapid global spread: in 1958, the AFT was reported from both Europe and tropical Africa; in 1975, from the USA; in 1981, from Mexico; and in 1999, from Brazil. Today, the AFT is present on all continents except Antarctica and is particularly widespread in North America, where it has been reported in 312 fish species and 11 non-fish hosts (Kuchta et al. 2018). In the Americas, AFT has a continent-wide distribution, with records spread from Canada, the United States, Mexico, Guatemala, Honduras, and Panama to Brazil and Argentina, with Mexico being the country with the highest number of records (Eiras et al. 2010; Salgado-Maldonado et al. 2015; Kuchta et al. 2018; Pérez-Ponce de León et al. 2018). According to Kuchta et al. (2018), the AFT is distributed in tropical, temperate, and subtropical climates, with a higher prevalence in subtropical regions, where approximately 50% of reported cases are associated with endemic fish with restricted distributions. This finding led to the untested hypothesis that native species are more vulnerable to AFT than their accepted natural hosts, cyprinid fishes (Scholz et al. 2012; Kuchta et al. 2018; Pérez-Ponce de León et al. 2018).

The AFT has been extensively co-introduced with its primary cyprinid hosts, common carp (*Cyprinus carpio*) and grass carp (*Ctenopharyngodon idella*), as well as through the unregulated distribution of minnows (common name for several genera of the family Cyprinidae) used as bait and of cyprinodontiform fishes such as the poeciliids guppy (*Poecilia reticulata*) and mosquitofish (*Gambusia affinis*) (Kuchta et al. 2018). The introduction of exotic fish can have important consequences for native species, not only through direct competition but also by co-introduction of their pathogens (García-Berthou et al. 2005; Smith et al. 2009; Hansen et al. 2013; Goedknegt et al. 2016). This is the case with the AFT, the causal agent of bothriocephalosis, which affects many fish species and represents the most devastating parasitic disease for the carp family (Scholz et al. 2012; Rubio et al. 2016; Ahmad et al. 2018; Dove and Fletcher 2000; Fu et al. 2022).

The primary signs of bothriocephalosis include gut necrosis and inflammation, abdominal distention, local hemorrhages, hemorrhagic enteritis, and gut epithelial desquamation. In severe cases, this disease also causes ulcers, epithelial compression (*e.g*., neoplasia), and intestinal blockade (Scholz et al. 2012). Diseased fish can also display poor sexual development and behavioral changes, such as laziness or lethargy, emaciation, anorexia, and gasping (Scholz et al. 2012; Ahmad et al. 2018). In general, AFT attracts much attention not only because of the ravages of the disease (particularly in farmed fish) but also because of its low host specificity, both for intermediate and final hosts.

Given the rapid geographical and hospedatorial expansion of the AFT—leading to its recognition as the most successful invasive parasite of freshwater fishes, despite its complex life cycle, its documented pathogenicity and apparent continued expansion and hence its potential impact in a changing world—, here, we review the knowledge on the AFT and propose new avenues for future research. We first present an overview of the parasite’s complex life cycle to highlight outstanding unresolved questions. We then present an updated, alternative history of the AFT’s dispersion in Mexico, which would better account for its nationwide distribution—whose ample and updated records we use to attempt to address some of the unanswered questions posed by Kuchta et al. (2018). Finally, we identify persisting knowledge shortfalls and suggest novel methods to try to elucidate them.

### Overview of the complex life cycle of *Schyzocotyle acheilognathi*

The genus *Schyzocotyle* (Neodermata: Cestoda: Bothriocephalidea) comprises parasites with complex life cycles and low host specificity, except for *Bothriocephalus rarus* (Jarroll 1979; Marcogliese and Esch 1989; Brabec et al. 2023). The life cycle of AFT has been described in detail elsewhere (e.g., Bush et al. 2001).

**Fig. 1:**
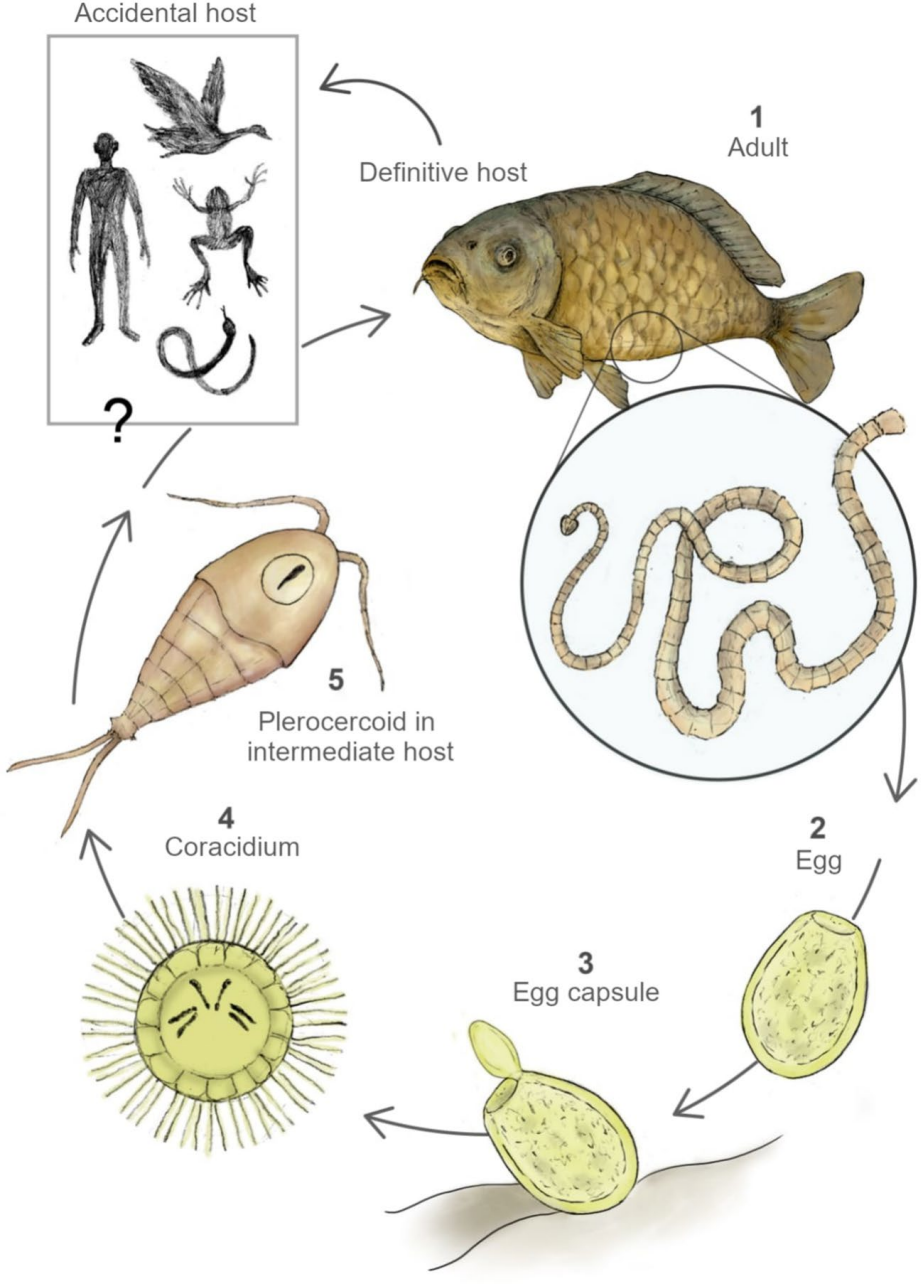
The Asian fish tapeworm (AFT) *Schyzocotyle acheilognathi* infects freshwater fish, causing bothriocephalosis. The life cycle of the AFT involves two hosts. **1** Gravid proglottids of the adult AFT release eggs which are eliminated with the feces; **2** eggs in the aquatic environment begin embryonic development at 28 to 30 °C in 1–5 days; **3** upon hatching, the larva takes on an oval shape and exhibits bending movements, allowing it to be expelled into the external environment; **4** the free-swimming larva, a six-hooked oncosphere or hexacanth, also called coracidium, takes on a spherical shape surrounded by cilia; **5** the coracidium penetrates the intestinal wall of the copepod intermediate host and migrates to the hemocoel, where it becomes an infective plerocercoid. A plerocercoid can potentially survive longer in copepods which enter dormancy or diapause. The life cycle is completed when a fish consumes infected copepods. Accidental transmission through ingestion of infected copepods has been demonstrated in other non-fish vertebrates. This figure is based on the research conducted by Kline et al. (2007)

Adult tapeworms inhabit the intestine of freshwater fish, where they release eggs into the water through the host’s feces (Fig. 1). Once hatched, the free-swimming larva (coracidium) consists of a ciliated embryophore enclosing a hexacanth oncosphere with six hooks (Scholz et al. 2012). The coracidium actively swims in search of copepod hosts, but if no host is encountered within a short period, it loses motility and dies.

Several copepods within the genera *Aconthocyclops*, *Cyclops*, *Macrocyclops*, *Megacyclops*, *Mesocyclops*, *Thermocyclops*, and *Tropocyclops* serve as intermediate hosts (see Marcogliese and Esch 1989; Horosheltseva et al. 2021). After ingestion, the larva penetrates the intestinal wall and develops within the hemocoel into a plerocercoid—the infective stage for fish (Körting 1975; Scholz et al. 2012). Currently, our understanding of the molecular mechanisms by which AFT invades copepods is limited. Although the morphological aspects of this transition are well characterized (Pool 1985), the cellular and molecular mechanisms underlying host invasion and development are poorly understood.

The cycle is completed when a fish ingests an infected copepod. Plerocercoids attach to the intestinal wall and mature into segmented adults within days to months, depending on temperature. In temperate regions, development is favored around 20 °C, and postcyclic transmission may occur under high host densities (Hansen et al. 2007). Adults reside in the midgut of fish, where it is common to find more than one cestode per host (Diaz-Castañeda et al. 1995).

The Asian fish tapeworm shows specificity for copepods as intermediate hosts and freshwater fish—mainly cyprinids—as definitive hosts. However, plerocercoids have occasionally been found in amphibians, reptiles, and birds, which act as accidental or mechanical hosts aiding parasite dispersal to new habitats (Prigli 1975; Scholz et al. 2021; Yera et al. 2013; Kuchta et al. 2018; Pérez-Ponce de León et al. 2018).

### Historical dispersion of AFT in Mexico

It is generally accepted that the first record of AFT in Mexico dates to 1981 (Kuchta et al. 2018), following the introduction in 1965 of grass carp, *C. idella*, to the Tezontepec Fish Center, in the state of Hidalgo, in central Mexico (Rosas-Moreno 1976). Once grass carp populations were established in Tezontepec in 1971, fish were translocated to southwestern Mexico, to the Fish Center at Lake Pátzcuaro and the El Infiernillo dam, both located in the state of Michoacán (López-Jiménez 1980; Osorio-Sarabia 1982; Salgado-Maldonado and Rubio-Godoy 2014). By 1972, grass carp was recorded to complete its life cycle in rivers draining to both the Gulf of Mexico and the Pacific, as well as in hatcheries, dams, and other water bodies (Bean 2008; Salgado-Maldonado et al. 2001a, b; García-López et al. 2016; Salgado-Maldonado et al. 2020). In 1977, Osorio-Sarabia recorded the first instance of AFT infecting *C. idella* at the El Infiernillo dam, while in 1981, it was recorded in the Tezontepec Fish Center, Hidalgo (López-Jiménez 1980; Osorio-Sarabia 1982). Regardless of whether 1977 or 1981 was the first valid record, it is quite surprising that AFT is now found throughout Mexico if the parasite was first introduced in 1965 with grass carp, which means an extremely rapid dispersal in a vast (1.973 million km^2^) and orographically complex territory within less than 60 years.

A probably more reasonable explanation is that the AFT was first translocated to the country with the common carp, *C. carpio*, which was exported from France to Mexico in 1872–1873 and introduced into the dams built in the northeast of the Mexican territory (Salgado-Maldonado and Rubio-Godoy 2014). Later, carps were scattered in estuaries, dams, oases, and wild environments through a rural fish farming program, which led to the overspill of AFT to the endemic ichthyofauna (Salgado-Maldonado and Rubio-Godoy 2014; Espinosa-Pérez and Ramírez 2015). Besides, the translocation and movement of native fish species in Mexico—driven by aquaculture and commercial activities following the introduction of exotic hosts—may have contributed to the spread of AFT. Although there is no direct evidence to link these movements to the parasite’s dissemination, this hypothesis is supported by the remarkable diversity of freshwater fish in Mexico (García-Prieto et al. 2022) and the high frequency of AFT records in commonly studied species, which are not cyprinids and hence not related to AFT’s primary hosts (Pérez-Ponce de León et al. 2018). These other hosts include species of the order Centrarchiformes, such as *Micropterus salmoides* (largemouth bass); Atheriniformes, including *Chirostoma estor*, *C. attenuatum*, and *C. grandocule*—collectively known as “charales” or “whitefish”—and *Atherinella balsana* (“Balsas silverside”), which are widely distributed across freshwater bodies in the states of Michoacán, Jalisco, Nayarit, Aguascalientes, Estado de México, and Guanajuato (Paulo-Maya 2000; Blancas et al. 2003). In addition, ornamental poeciliid fish species such as guppies and mollies from the genus *Poecilia*, swordtails and platies from *Xiphophorus*, and mosquitofish from the genera *Gambusia* and *Poecilia* have been widely introduced in various regions of the country, either through the aquarium trade or biological control programs (Mar-Silva et al. 2021). AFT has been reported in several of these translocated poeciliids, including *Poecilia reticulata*, *P. mexicana*, *Gambusia affinis*, *G. holbrooki*, *Xiphophorus hellerii*, and *Pseudoxiphophorus bimaculatus*, suggesting that their translocation may have facilitated the parasite’s dissemination. The presence of AFT has also been confirmed in native and endemic non-cyprinid fishes such as *Pseudoxiphophorus jonesii*, *Xiphophorus maculatus*, the goodeid *Goodea atripinnis*, and threatened species of the genus *Chirostoma*, including *C. jordani* and *C. lucius* (see Salgado-Maldonado 2006; Aguilar-Aguilar et al. 2008; Quiroz-Martinez et al. 2013; Salgado-Maldonado and Quiroz-Martinez 2013).

Despite these records, no specific studies have yet been conducted in Mexico to evaluate how the intra- and interregional movement of native species may have influenced the dispersal of this cestode. In contrast, research focused on other parasitic groups—such as gyrodactylids—has explored interactions between introduced and native species. For instance, while Rubio-Godoy et al. (2016) found no evidence of gyrodactylid parasites transferring from invasive poeciliids to the endemic goodeid *Goodea atripinnis*, García-Vásquez et al. (2017, 2021) documented the overspill of the tilapia pathogen *Gyrodactylus cichlidarum* to both native cichlids and several poeciliid fishes. These findings emphasize the value of assessing parasitic risks associated with the co-occurrence of native and exotic species.

These and other transfaunation-related events, potentially dating to the end of the XIX^th^ century, led to Mexico becoming the nation with the highest number of AFT records, a situation likely arising from a combination of favorable environmental conditions and the high biodiversity of its ichthyofauna—hence, the availability of potential hosts (Salgado-Maldonado and Pineda-López 2003; García-Prieto et al. 2022).

To contextualize the AFT’s successful distribution across the nation, one must understand that Mexico encompasses a vast area and presents intricate geographic conditions where the Nearctic and Neotropical biogeographical provinces intersect, facilitating the coexistence of species with diverse evolutionary backgrounds (Halffter and Morrone 2017). The registered ichthyofauna in Mexico includes more than 500 native and endemic fish species, making it one of the most ichthyologically diverse countries in the world (Lyons et al. 2020; Miller et al. 2005). In Mexico, approximately 110 species of freshwater fish are known hosts of AFT, of which approximately 36 are endemic species (Pérez-Ponce de León et al. 2018), including Cypriniformes and Cyprinodontiformes, as well as several evolutionarily distantly related native fish species (Choudhury and Cole 2012; Kuchta et al. 2018; Pérez-Ponce de León et al. 2018). Moreover, AFT has been recorded in all states of Mexico, except Nuevo León (Salgado-Maldonado and Rubio-Godoy 2014; Pérez-Ponce de León et al. 2018; García-Prieto et al. 2022), with a higher prevalence in the center and south of the country (for more details, see Peréz-Ponce de León et al. 2018; García-Prieto et al. 2022). Despite AFT not being documented in Nuevo León, its presence cannot be discounted, as it has been reported from *Notropis braytoni*, a species native to the Rio Grande in the USA and the Rio Conchos drainages, in the neighboring Mexican states of Chihuahua, Coahuila, and Tamaulipas (Choudhury et al. 2004; Bean 2008; Bean and Bonner 2010).

For all the above, we consider Mexico to be an appropriate natural laboratory for assessing the ecological and environmental variables that correlate with the dispersion of AFT.

### Old and new questions regarding the Asian fish tapeworm

Several studies have been conducted on the biology of *S. acheilognathi;* for example, describing its origin, taxonomy, and phylogeny (Scholz et al. 2012; Brabec et al. 2015; 2016); its distribution (Salgado-Maldonado 2006; Kuchta et al. 2018; Pérez-Ponce de León et al. 2018; Palermo et al. 2021a,b) and control (Kline et al. 2009; Mitchell and Darwish 2009; Iles et al. 2012); its microbiota (Fu et al. 2022; Casanova-Hernández et al. (n.d.) *in press*); its virulent capacity under different geographical scenarios and its economic and productive impacts (Scholz et al. 2012; Pérez-Ponce de León et al. 2018); and its genetic diversity (Luo et al. 2002; Brabec et al. 2018; Pérez-Ponce de León et al. 2018; Villa-O'Dogherty et al. 2025). Despite these efforts, significant knowledge gaps remain, such as the full characterization of the primary ecological and intrinsic drivers that enable AFT’s wide geographic distribution and low host affinity.

In their comprehensive review, Kuchta et al. (2018) proposed several unanswered questions regarding AFT. These included 1) establishing the original geographic provenance of the parasite; 2) determining the genetic variation between AFT collected from different fish populations/host species/geographic locations; 3) assessing the thermal preference/tolerance of the parasite and establishing whether this is related to its ancestral (source) geographic populations; 4) analyzing whether AFT’s genetic variation is related to its invasive success; 5) determining the parasite’s impact on native and ornamental fishes; and 6) evaluating the impact of climate change on this parasite and how it affects its spread. Here, we reviewed the latest advances on these outstanding questions (except the first one, for which no information is available) and used data recently compiled in Mexico to address these questions, to figure out if they have been answered (albeit partially), analyze if they are still relevant, and propose avenues to address them. We also suggest new lines of research on this topic, particularly using ecological and molecular approaches*.*

### Data search

In this review, we surveyed the available information on *S. acheilognathi*, including data related to its currently reported hosts, as well as their geographic distribution area, life cycle, population genetics, and microbiota. Several data sources were reviewed, including the Web of Science, NCBI, Scopus, and Helminthological Abstracts, using the keyword “bothriocephalosis” and all synonyms for *Schyzocotyle acheilognathi* (for a complete list of synonyms, see WoRMS 2025). We used keywords such as "bacteria and parasite," "gut microbiota in fish," "microbiota in parasites," and “endoparasitic symbiont” to retrieve information on bacterial taxonomic units. We also retrieved information on host-parasite interactions, including "metabolomics and parasite and fish," "bacteria-parasite metabolism," "proteomics and parasite and fish," and "transcriptomics and parasites." We also consulted dissertation material available on the official pages of public and private educational institutions. The Global Biodiversity Information Facility (GBIF 2024) database was also explored to identify the areas with the largest number of known records for the Asian tapeworm, to focus the search for more specialized databases to explore the broad-scale ecological behavior of this parasite. We sorted the information by topic, based on its relevance. The database compiled by García-Prieto et al. (2022) was used to conduct an exploratory assessment of the impact of infection contrasting native and non-native species. To avoid underestimation of parasitological values, ecological parameters were retrieved from previously reported host species, selecting only those studies that included more than 15 individuals per species, in accordance with the criterion established by Vidal-Martínez et al. (2001). This selection ensured the reliability of the estimates for three fundamental parasitological metrics: prevalence, mean intensity, and abundance (Marques and Cabral 2007). Parasitological records were compiled for a total of 114 fish host reports, of which 12 corresponded to non-native species and the remainder to native fish. Only records that reported all three parasitological metrics—prevalence, intensity, and abundance—were included.

To investigate whether there was a differential parasitic effect between native and exotic hosts, the k-prototypes algorithm was applied. This clustering technique is appropriate for mixed datasets that include both numerical variables (*e.g*., parasitological metrics) and categorical variables (*e.g*., host classification as native or non-native), as described by Madhuri et al. (2014). Additionally, the García-Prieto et al. (2022) database was used to map the distribution of the AFT in Mexico and it was employed to assess the ecological and environmental variables potentially influencing its success and distribution, with a particular focus on thermal preferences, as discussed by Kuchta et al. (2018).

## Results/discussion

1. **Establishing the original geographic provenance of the Asian fish tapeworm**

Traditionally, Asia has been considered the region of origin of the AFT, largely due to its association with cyprinid fishes, such as *C. carpio*, which are widely distributed across the continent. However, records show that AFT existed in tropical African regions before the human-mediated introduction of Asian fishes (Kuchta et al. 2018), challenging the hypothesis of a strictly Asian origin. These findings suggest that the evolutionary history of AFT is older and more complex than previously assumed, prompting a reevaluation of whether its spread beyond Asia could have occurred through natural means rather than solely through anthropogenic mediation. However, this will not be addressed here, as we provide no data to contribute to this topic.

2. **Determining the genetic variation between AFT collected from different fish populations/host species/geographic locations**

The genetic variation of AFT reflects a complex evolutionary scenario, characterized by considerable intraspecific heterogeneity associated with both geographical and ecological factors (Villa-O'Dogherty et al. 2025). As a cosmopolitan and generalist parasite, its colonization of diverse hosts and environments has likely promoted genetic differentiation among populations. Early molecular studies reported high divergence between Asian and African lineages (Luo et al. 2002; Brabec et al. 2018), suggesting restricted gene flow likely driven by independent introduction histories, spatial isolation, or host-related selection. Recent evidence from Mexican populations confirms this trend, identifying four genetically structured groups differing in allelic richness and private alleles (Villa-O'Dogherty et al. 2025).

However, the ecological meaning of this differentiation remains unclear. The apparent structure could reflect sampling bias, founder effects, or methodological artefacts rather than true adaptive divergence—a common issue in invasive taxa with complex life cycles (Lee 2002; Roman and Darling 2007; Brabec et al. 2016). Moreover, while some evidence suggests host-associated differentiation (Luo et al. 2002), the influence of host identity appears secondary to geographic isolation, as observed in other generalist helminths such as *Gyrodactylus salaris* and *Diplostomum spathaceum* (Meinilä et al. 2004; Blasco-Costa et al. 2014).

The heavy reliance on ribosomal internal transcribed spacer (ITS) markers further complicates interpretation due to paralogous copies that may generate spurious signals (Brabec et al. 2016). Consequently, the integration of mitochondrial, single-nucleotide polymorphisms (SNPs), and genome-wide datasets is critical for resolving true evolutionary patterns. Similar transitions toward high-resolution genomic tools have transformed our understanding of invasive parasites such as *Angiostrongylus cantonensis* (Červená et al. 2019) and highlight the potential of omics to disentangle genetic connectivity and adaptive mechanisms in AFT (McVeigh 2020; McGaughran et al. 2024).

Observed heterozygote deficits and deviations from Hardy–Weinberg equilibrium across populations could reflect founder events, self-fertilization, or the Wahlund effect—processes typical of species undergoing rapid range expansion. Yet, the paradox remains: how can such low variability sustain broad ecological success? This apparent contradiction suggests that plasticity, epigenetic modulation, or host-mediated metabolic complementarity may buffer against the constraints of limited genomic diversity (Dybdahl and Kane 2005; Prentis et al. 2008; Lymbery et al. 2020).

Ultimately, integrative population genomics and transcriptomics offer the most promising route to determine whether AFT*’s* success relies on genomic innovation, host-driven plasticity, or both. Such approaches will be vital to assess the evolutionary and epidemiological consequences of this parasite’s ongoing expansion worldwide.

3. **Analyzing whether AFT’s genetic variation is related to its invasive success**

Studies on the genetic variation of AFT have provided valuable insights into the mechanisms underlying its remarkable invasive success. Using molecular tools—primarily sequencing of ribosomal ITS—researchers have reported low genetic diversity across both native and introduced populations (Villa-O'Dogherty et al. 2025). Paradoxically, this limited variability does not appear to constrain its ability to establish and thrive across heterogeneous aquatic environments worldwide. Similar paradoxes have been described in other invasive taxa, where phenotypic plasticity, broad ecological tolerance, and flexible adaptive responses compensate for low genomic variation (Dybdahl and Kane 2005; Prentis et al. 2008; Lymbery et al. 2014, 2020).

An early indication of possible genetic structuring in AFT was reported by Luo et al. (2002), who detected differences between strains infecting distinct hosts—potentially reflecting host-driven divergence. However, such interpretations must be approached with caution, as paralogous copies in nuclear ribosomal genes can obscure true phylogenetic relationships (Brabec et al. 2016). To better resolve the parasite’s evolutionary potential, expanding beyond ribosomal markers toward mitochondrial genes (e.g., cox1, COI), complete mitogenomes, and genome-wide datasets is essential. Comparable genomic approaches in other invasive parasites have illuminated mechanisms by which low diversity coexists with strong invasion capacity (Červená et al. 2019; Robinson et al. 2018; Biedrzycka et al. 2020). For example, the nematode *Angiostrongylus cantonensis* exhibits strikingly low mitochondrial diversity across distant regions yet continues to expand globally (Červená et al. 2019). Similarly, the signal crayfish *Pacifastacus leniusculus* demonstrates that genetic structure and host–parasite dynamics can facilitate establishment even under reduced genetic variability (Robinson et al. 2018). Moreover, some studies indicate that invasion potential may rely more on standing genetic variation at immune loci than on overall genomic diversity (Biedrzycka et al. 2020).

In AFT, the ability to infect phylogenetically distant hosts and complete its life cycle under contrasting environmental conditions suggests that mechanisms beyond classical genetic diversity—such as adaptive gene expression, epigenetic regulation, or metabolic complementarity with hosts—may underlie its invasive success. Although these hypotheses remain to be empirically tested, they align with recent transcriptomic findings in other parasitic taxa showing host-dependent modulation of gene expression and metabolic pathways (Wang et al. 2020; Wendt et al. 2020; Buddenborg et al. 2023).

This apparent paradox between low genetic diversity and high invasiveness raises several key questions: Could AFT’s success be primarily driven by plasticity and epigenetic regulation rather than adaptive genomic variation? To what extent has its genome undergone erosion or streamlining because of host dependency? Do different host–parasite combinations drive subtle transcriptomic or metabolic adaptations? How strong is the global genetic connectivity among populations, and does it facilitate rapid colonization of new regions?

Addressing these questions through integrative omics approaches could reveal whether AFT’s invasion success depends mainly on genomic innovation, host-mediated plasticity, or the evolutionary resilience characteristic of many generalist parasites.

4. **Determine the parasite’s impact on native and ornamental fishes**

AFT has been documented as highly pathogenic to a wide variety of freshwater fish species, both native and exotic. Reports suggest that its impact may be particularly severe on native species, possibly due to the lack of a co-evolutionary history with the parasite or to specific ecological conditions that increase host susceptibility. For instance, Choudhury et al. (2004) reported that native species in Arizona’s Little Colorado River exhibited higher infestation rates and more pronounced physiological effects compared to introduced species. Similarly, Palermo et al. (2021a, b) warned that detection of AFT in endemic fish from Western Australia may pose a significant conservation threat.

Although direct evidence of the impact of AFT at the population level in natural environments remains limited, multiple studies documenting the parasite’s introduction into new areas support well-founded concerns regarding its invasive potential (Dove and Fletcher 2000; Heckmann 2000, 2009; Salgado-Maldonado and Pineda-López 2003). Estimates of ecological parameters such as prevalence, infection intensity, physiological disruptions, and body condition deterioration have been instrumental in hypothesizing potential adverse effects on particular host species (Nie and Hoole 2000; Hansen et al. 2006; Zargar et al. 2012).

On the other hand, different ornamental fish have frequently been documented as hosts of AFT, with outbreaks reported in cultured systems of species such as guppy, *P. reticulata*, southern platyfish, *X. maculatus* (Evans and Lester 2001; Kuchta et al. 2018; Assis and Pinto 2024), koi carp, *C. carpio* (Han et al. 2010) and convict cichlid, *Amatitlania nigrofasciatus* (Matey et al. 2015). However, this category (*i.e*., ornamental fish) is problematic from an ecological perspective, as classifying a fish as “ornamental” reflects purely anthropocentric interests and holds no biological relevance for the host-parasite interaction, which infects their host regardless of aesthetic or commercial considerations. The only obvious value of including ornamental fish in this discussion lies in their role as highly effective vectors for parasite dispersal, facilitated by international trade and the long-distance movement of live fish. Although the physiological impact of the parasite on ornamental fish species has rarely been evaluated experimentally, there is evidence of severe effects such as protein depletion, digestive enzyme disruption, muscle fatigue, and juvenile mortality (Liao and Shih 1956; Davydov 1978; Granath and Esch 1983b; Hansen et al. 2006). Documented cases such as infected red discus, *Symphysodon discus* (Kosuthová et al. 2015) and the high prevalence of the AFT in baitfish stores in the US (Boonthai et al. 2017) underscore that, rather than focusing on artificial categories like “ornamental,” the core issue lies in human-mediated movement of organisms and their role in facilitating parasite spread to new environments. So we decided to reduce Kuchta’s et al. (2018) original question to more natural categories and simply assign the fish to native versus non-native hosts. For the sake of simplicity we used data compiled by García-Prieto et al. (2022) to explore whether there were significant differences in parasitological parameters between these two ecological groups. We applied the k-prototypes algorithm, designed to handle mixed-type data, including both numerical variables (e.g., prevalence, mean intensity, abundance) and categorical ones (native vs. non-native host status), to identify meaningful clusters or patterns within the dataset and better understand the dynamics or potential groupings of the parasite AFT associated with different host orders, as it has been documented in the literature that some families, particularly smaller-sized fish, are more vulnerable (Pérez-Ponce de León et al. 2018).

**Fig. 2:**
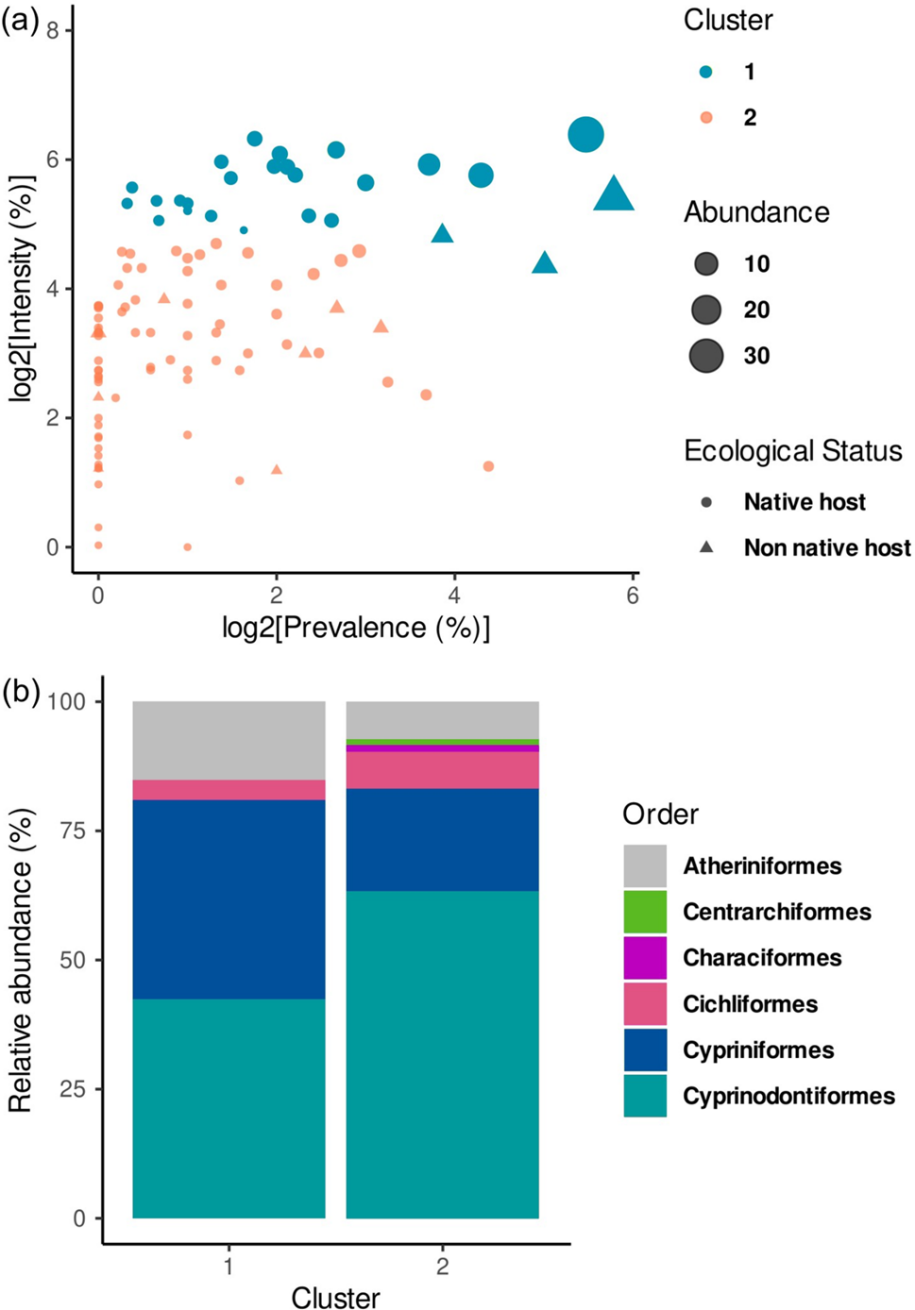
Clusters of Asian fish tapeworm (AFT) *Schyzocotyle acheilognathi* hosts based on infection metadata associated with the fish hosts (including prevalence, intensity, and abundance) using k-Prototypes algorithm. Panels (**A**) and (**B**) show the clusters and distribution of *S. acheilognathi* infection data based on the ecological parameters of fish host species in Mexico, as well as the proportion of infected species in taxonomic order. Two main host clusters were identified, comprising both native and non-native species, and differentiated by their ecological characteristics. Panel (**A**): Cluster 1 includes hosts with variable parasitic intensity and mean relative abundance, ranging from low to high, while Cluster 2 includes fish with low prevalence and moderate intensity. Panel (**B**): All represented taxonomic orders act as hosts of the parasite, though with differing levels of mean relative abundance. Cyprinodontiformes contain the highest number of affected species, followed by Cypriniformes (including the parasite’s natural hosts), Atheriniformes, Cichliformes, Characiformes, and Centrarchiformes

Cluster analysis revealed two main groups of hosts, distinguished by their susceptibility to the parasite, as shown in Fig. 2. Both groups included native and non-native fish species. The first cluster exhibited low to moderate average values across the evaluated ecological parameters, while the second cluster displayed a broader range of prevalence values—from low to high—with prevalence ranging from 23.1% to 59.57%, mean intensity between 1.15 and 55.1, and mean abundance from 0.17 to 23.31.

Both native and non-native species tended to harbor variable parasite loads, likely influenced by external factors such as disease seasonality and geographical and environmental conditions (e.g., temperature). Moreover, biological traits of the definitive host, such as trophic level (e.g., herbivore, carnivore, insectivore, benthivore, etc.), may also influence infection dynamics, as reported in previous studies (Hansen et al. 2007), but to a lesser extent.

The second group, which includes a mix of native and non-native species, exhibited high prevalence and moderate intensity, suggesting that certain species — particularly small-sized fish — could act as efficient reservoirs of the parasite, contributing to the persistence of the biological cycle of infection. Furthermore, some hosts, despite showing lower values in ecological parameters considered, may represent optimal hosts for supporting high parasite loads (Salgado-Maldonado and Pineda-López 2003; Zargar et al 2012).

Overall, these findings indicate that the host preference of AFT does not appear to be taxonomically constrained. Its infection patterns seem to be independent of phylogenetic distance or the native/non-native status of the host, challenging previous hypotheses suggesting stricter host specificity (Kuchta et al. 2018; Pérez-Ponce de León et al. 2018). Nevertheless, this analysis has limitations, including small sample sizes for certain taxonomic groups, variability among data sources, and the lack of information on intermediate vectors—particularly copepods—which are critical components in the parasite’s life cycle.

**Fig. 3:**
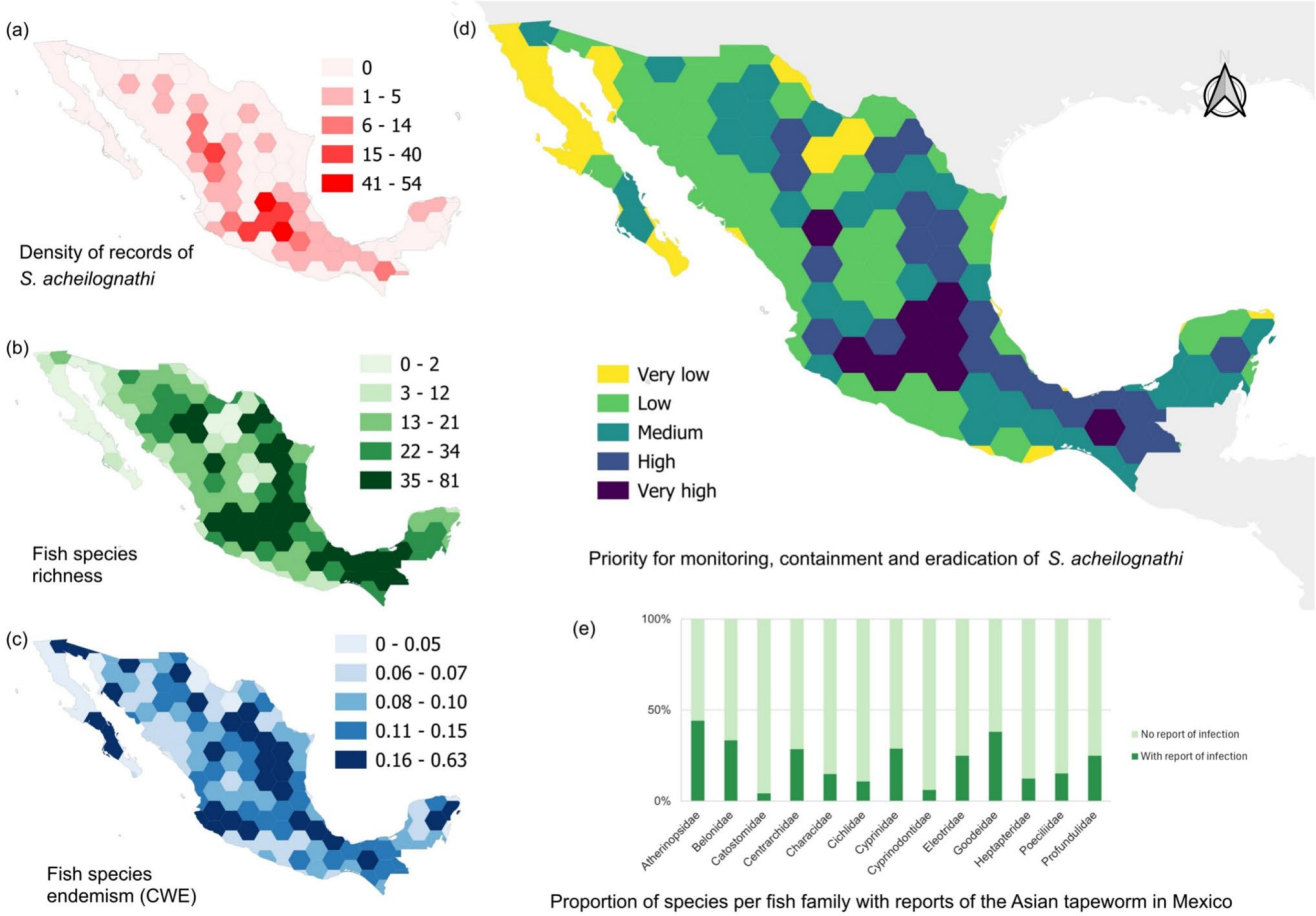
Maps of freshwater fish endemism and biodiversity in Mexico, compared with infection records of Asian fish tapeworm (AFT) *Schyzocotyle acheilognathi* to identify priority conservation areas. The maps highlight regions with high freshwater fish richness and endemism, overlaid with documented cases of *S. acheilognathi* infection. The panels include (**a**) density of *S. acheilognathi* records; (**b**) fish species richness; (**c**) fish species endemism; (**d**) priority areas for monitoring, containment, and eradication of *S. acheilognathi*; and (**e**) proportion of species per fish family with reports of AFT infection in Mexico. These maps reveal areas of potential concern for parasite-host interactions and inform conservation strategies

Although AFT exhibits a broad host range, certain species and regions—especially those with high freshwater fish richness or endemism—may be particularly vulnerable. Thus, we generated endemism and biodiversity maps of freshwater fish species and compared them with infection records to identify priority areas for conservation (Fig. 3). With this evidence, we next considered that sites with more species may be more susceptible to invasions of this parasite since they have a greater availability of potential hosts; likewise, regions with the presence of endemic species are considered vulnerable due to the risk of loss of diversity. A third factor considered for the prioritization of actions relates to localities where AFT is already well established. With these three factors in mind, we generated a proposal of priority areas for monitoring, managing, and containing AFT in Mexico. For the division of sites, a hexagonal grid with a spacing of 1.4 degrees was created, wherein for each polygon, the following values calculated from the databases of García-Prieto et al. (2022) and CONABIO (2024) were averaged in a normalized way: 1) density of records of *S. acheilognathi*; 2) number of native freshwater fish species; and 3) Corrected Weighted Endemism (CWE) of the fish species. We also calculated the percentage of known AFT-parasitized species in each fish family.

According to our proposal, central Mexico, along with particular areas in the northeast and southeast, are the regions with the highest priority for monitoring the invasion status of AFT. However, in practice, most regions of the country exhibit a high priority due to the widespread presence of the parasite and the significant number of existing endemic species. Of the fish families in which the AFT has been recorded, Atherinopsids stand out, as there are infection records for almost half of the species recorded for the country (n = 15, 44%); a similar situation occurs in the fish families Goodeidae (n = 16, 38%) and Cyprinidae (n = 24, 28%). Mexican law recognizes nearly all species in these families as endangered (SEMARNAT 2025). Even in families with fewer endangered species, like Profundulidae, the only two species on the list have been infected by AFT.

5.  **Assessing the thermal preference/tolerance of the parasite, and establishing whether this is related to its ancestral (source) geographic populations **

In view of the AFT’s widespread distribution, we consider this species as a suitable candidate to implement ecological niche modeling (ENM) approaches, which can help to indirectly estimate the tolerance and suitability of this parasite to different environmental variables or to detect geographic areas prone to invasions. ENM aims to determine a parasite’s geographic distribution, understand its transmission dynamics, and predict new intermediate hosts or reservoirs. ENM approaches have improved understanding of the potential distribution of parasites and diseases, even when the effects of infection/pathology are unclear (Maher and Timm 2014; Samy et al. 2014; Blackburn et al. 2017; Johnson et al. 2019). ENM has also been highly useful to estimate the behavior of different zoonotic diseases (Lawrence et al. 2023), where one of the main factors to consider is the ecological niche of the vector. However, these approaches have been much less employed to study parasites.

The main limitation of using ENM for parasites is that it is (usually) reduced to niche modeling for their hosts, which is a true representation of the parasite’s niche if the parasite is highly specialized and present throughout its host distribution. However, as commented previously, AFT can infect more than 100 fish species, so we confidently assume that its ecological niche (in Grinellian terms) extends beyond that of its hosts. A semantic issue surrounding the concept of ecological niche has possibly slowed down the implementation of ENM methods for fish parasites. For example, Rohde and Rohde (2005), taking the classic Hutchinsonean definition of the multidimensional hypervolume of biotic and abiotic environmental variables, scrutinized the potentially relevant variables mentioned above, as well as any others related to the host, such as specificity, microhabitats (understood as the area of the host where the parasite thrives), macrohabitats of the host (this could be close to something abiotic, but being conditioned to the host, it is not), etc. In this sense, a pertinent question is whether AFT responds to broad-scale environmental variables in the same way that their hosts do.

To determine and define the geographic range of a parasite, it is necessary to know its life history, which includes the actual and potential intermediate and definitive hosts used by the parasite (Haverkost et al. 2010; Lira‐Noriega and Peterson 2014; Valdez-Espinoza et al. 2025). Predictive models allow the inclusion of ecological and environmental (abiotic) variables that may be related to life history. Additionally, to predict the further impact of AFT, these abiotic variables may help to understand not only its associated biological variables but also its geographic distribution range, including areas where it has successfully established and areas where it has not been found yet. Predictive models could also identify suitable habitats and the distribution of hosts, which may be limited by the absence of necessary hosts in its life cycle or by the presence of a definitive host that is incompatible with the intermediate host.

**Fig. 4:**
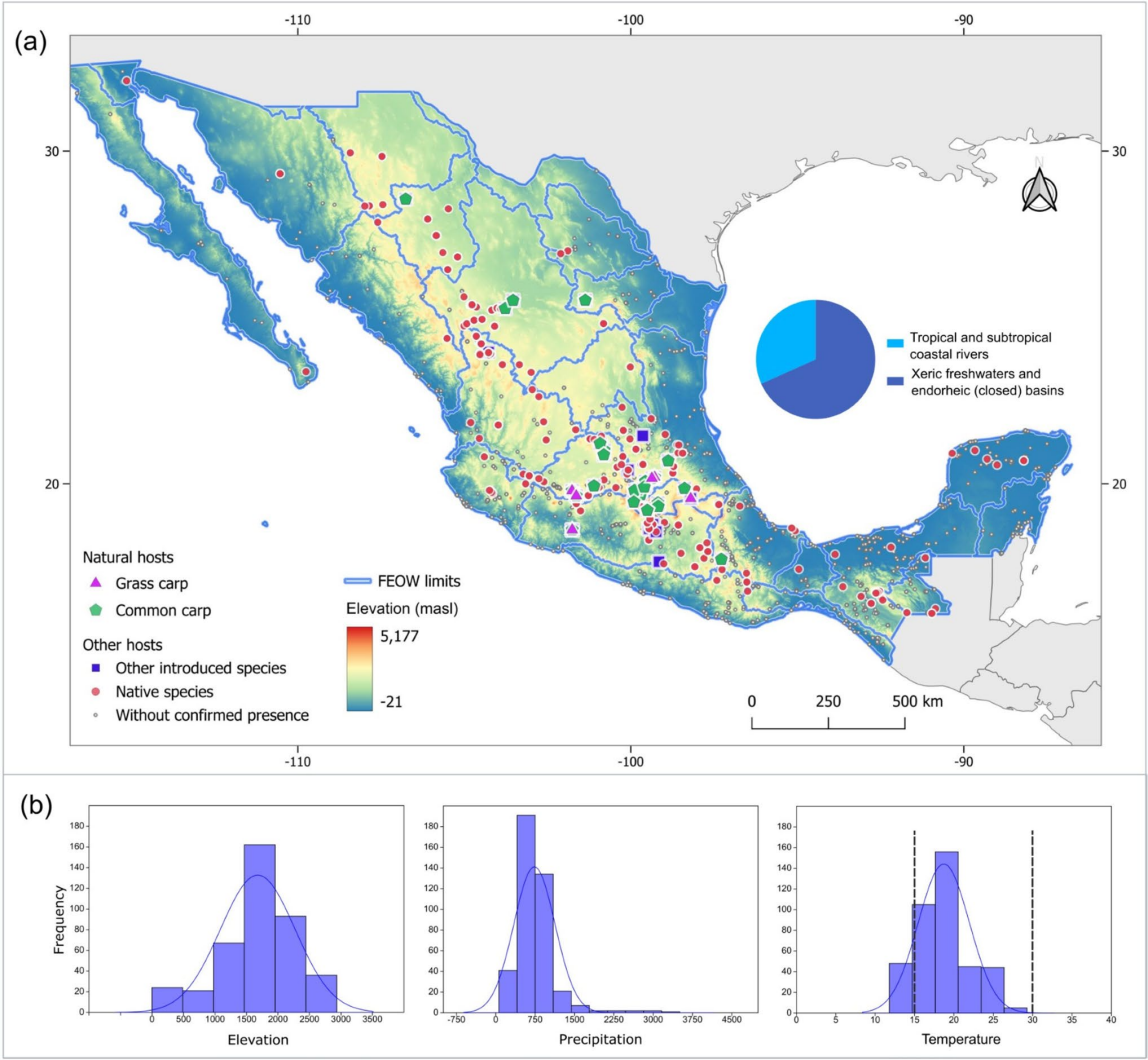
Distribution map of the fish host species infected with *Schyzocotyle acheilognathi* in Mexico. (**a**) Locations where the cestode was recorded infecting different fish hosts are shown as colored figures: grass carp *Ctenopharyngodon idella* (pink triangle); common carp *Cyprinus carpio* (green pentagon); other introduced fish species (blue square); and native fish species (red dot). Gray dots show locations where fish were sampled and no *S. acheilognathi* was found, as recorded in García-Prieto et al. 2022. Geographic regions shown include tropical and subtropical coastal rivers, xeric freshwaters, and endorheic (closed) basins. FEOW = Freshwater Ecoregions of the World. (**b**) Graphical representation of the distribution of macroecological and environmental variables for localities where AFT was recorded

To analyze the foregoing, we investigated whether there are associations between the presence of the Asian tapeworm and environmental variables that are likely relevant to freshwater fish and could have a detectable effect on a large scale. To assess this, we consulted the database of georeferenced records of helminth fish parasites in Mexico (Garcia-Prieto et al. 2022), which considers elevation, precipitation, and average temperature for each site (Fig. 4). We found that for each of these variables, the number of records for AFT follows a pattern that resembles a normal distribution, which would be the expected biological response. Importantly, this pattern does not seem to be biased by collection efforts, since proportionally, the highest number of overall parasite records for this country are found at elevations where the Asian tapeworm is rather scarce. In addition, the temperatures reported at a general level for this species (ranging from 15 to 30 °C) already differ if one looks at the sites where they have been recorded. The classification of Freshwater Ecoregions of the World (TNC and WWF 2019) also shows a clear geographic pattern in terms of biogeographic regions, with the highest number of AFT records concentrated in endorheic basins and xeric freshwaters. This pattern may arise from both environmental factors and the presence of hosts that are susceptible to parasitization.

Some authors suggest that river basins in the north of Mexico are not suitable for the growth and establishment of the cestode and that Nearctic fish species that inhabit these areas, particularly ictalurids and centrarchids, are not hosts of AFT (Pérez-Ponce de León et al. 2018). However, the records of *S. acheilognathi* in the southern United States do not seem to support this hypothesis (Choudhury et al. 2004; Bean 2008; Bean and Bonner 2010). Therefore, to get a rough idea of bioclimatic variables related to the success and distribution of AFT, it would be advisable to investigate disease reports and incidences in both the northern, Nearctic, and the southern, Neotropical parts of Mexico using predictive models.

6. **Evaluating the impact of climate change on this parasite’s further spread**

The successful dispersion of AFT has been determined by a combination of biological and environmental factors (Kuchta et al. 2018), with climatic conditions being a key factor in various regions of the world.

In the case of AFT, both temperature and precipitation seem to be key factors influencing its ability to colonize different hosts. Temperature affects both the infection rate and the development speed of infective stages (Davydov 1978; Granath and Esch 1983b; Oskinis 1994). Other environmental factors, such as humidity and water composition (particularly salinity, pH, and dissolved oxygen concentration), also influence the viability of infective larval stages (Granath and Esch 1983a; Emde et al. 2016). Furthermore, interactions with other organisms, including microorganisms and competitors, can modify the parasite’s ability to colonize its hosts (Marcogliese 2008; 2016; Mideo 2009). Host behavior, such as habitat preferences and immune responses, also influences the ability of AFT to establish itself in them (Fu et al. 2022).

In aquatic environments such as the cooling reservoirs of North Carolina, community dynamics and the interaction of AFT with its hosts are strongly conditioned by environmental variables. It has been observed that changes in water temperature, generated by human activity, can alter both the survival of the parasite and the host responses, directly affecting the prevalence of the infection and the distribution of the parasite among host fish (Budria and Candolin 2014; Biswas and Pramanik 2016; Giari et al. 2022). Additionally, studies on the suprapopulation dynamics of AFT in these reservoirs have shown how the abundance, dispersal, and prevalence of this parasite fluctuate in response to environmental and ecological factors, highlighting its ability to adapt to changing environmental conditions (Bush et al. 2001).

Previous research has documented that water temperature has a profound impact on the success and transmission dynamics of AFT. Riggs and Esch (1987) found that temperatures below 18 °C negatively affect the transmission of this parasite, while temperatures above 25 °C can decrease its transmission capacity. These results suggest that there is an optimal temperature range for the spread of this parasite, beyond which transmission is affected (Riggs 1986). Our analysis of environmental variables in Mexico indicated that factors such as precipitation, temperature, and elevation are crucial for the dispersal of AFT and that environmental attributes are closely related to the prevalence of this parasite (Fig. 4). Although no formal ecological niche model was developed in this study, the identified climatic and elevational patterns provide a valuable framework for future predictive analyses of the biogeography of the AFT. Similar approaches have been used to anticipate the distribution of other parasitic species under changing environmental conditions (Pickles et al. 2013; Chalghaf et al. 2018; Flores-López et al. 2022; Lu et al. 2022; Valdez-Espinoza et al. 2025).

Climate change is exacerbating the spread of parasitic diseases around the world, which justifies the relevance of investigating how climatic conditions influence the dispersal of AFT. However, further research is required to fully comprehend how climate change may modify the spread of parasites in the future.

### Other likely important but understudied determinants of the incidence of AFT

Other factors that may significantly explain AFT’s success have not received enough attention. This is the case with copepods, which, despite their critical role in AFT’s life history, have insufficient current data to determine the extent to which they influence the incidence of this parasite, as well as whether or not AFT prevalence is linked to copepod availability. For example, it is known that copepods from the Cyclopoida get into dormancy and diapause in response to unfavorable conditions (Dahms 1995; Hairston et al. 2000; Frisch 2002). Under these conditions, infested copepods can provide a safeguard for the parasite and when conditions are favorable, they come out of dormancy and complete their life cycles, facilitating the transmission of the cestode to its final host.

Because copepods are important food sources for small fish, it is likely that this phenomenon explains why the AFT’s lethality appears to have a strong ontogenetic component, particularly affecting smaller fish, and highlights the importance of a detailed analysis of the diet shift as a major factor influencing the likelihood of AFT infestation events. For instance, *Chirostoma* shift diets as they mature into adults, depending on the availability of food and the season of the year (Moncayo-Estrada et al. 2007). In the larval stage, these fish consume small insects and crustaceans, as well as other benthic organisms. As juveniles, they are carnivore generalists feeding on chironomids and zooplankton like ostracods, cladocerans, and copepods, but when they become adults, they become more ichthyophagous while also consuming insects, crustaceans, and microcrustaceans like amphipods and decapods (Moncayo-Estrada et al. 2007).

In addition, despite the detailed information regarding the morphological changes occurring in AFT during its life cycle, much less is known about the molecular events behind them. In this sense, the use of omics approaches could be relevant to research basic questions on the evolutionary biology and life cycle traits of AFT, as has been demonstrated for other parasites. For example, Buddenborg et al. (2023) conducted a comprehensive transcriptomic analysis of *Schistosoma mansoni*, covering eight developmental stages from eggs to adult worms. Similar omics-based approaches have revealed fine-scale molecular processes in this species (Díaz-Soria et al. 2020), demonstrating that RNA sequencing can yield detailed insights into parasite biology and functional genomics.

Based on the above, several questions emerge: What is the host range of AFT among copepods? To what extent does the distribution of copepod hosts determine the parasite’s geographical range? And how closely are copepod assemblages linked to the ecological success of *S. acheilognathi*? Addressing these questions requires studying the composition of copepod communities coexisting with parasitized fish, as this information may help relate disease incidence to the availability of intermediate hosts. Traditional taxonomic approaches remain valuable for identifying these communities, but their resolution can be greatly enhanced using high-throughput sequencing tools such as environmental DNA (eDNA) metabarcoding or shotgun sequencing (Taberlet et al. 2012; Shokralla et al. 2014; Blackman et al. 2019). Although these methods are now widely applied to assess marine copepod community assemblages (e.g., Yeh et al. 2020; Becker et al. 2021), they remain underutilized in freshwater systems, despite clear advantages such as reduced sampling effort and faster community characterization (Thomsen and Willerslev 2015; Andruszkiewicz et al. 2017; Blackman et al. 2019).

**Fig. 5:**
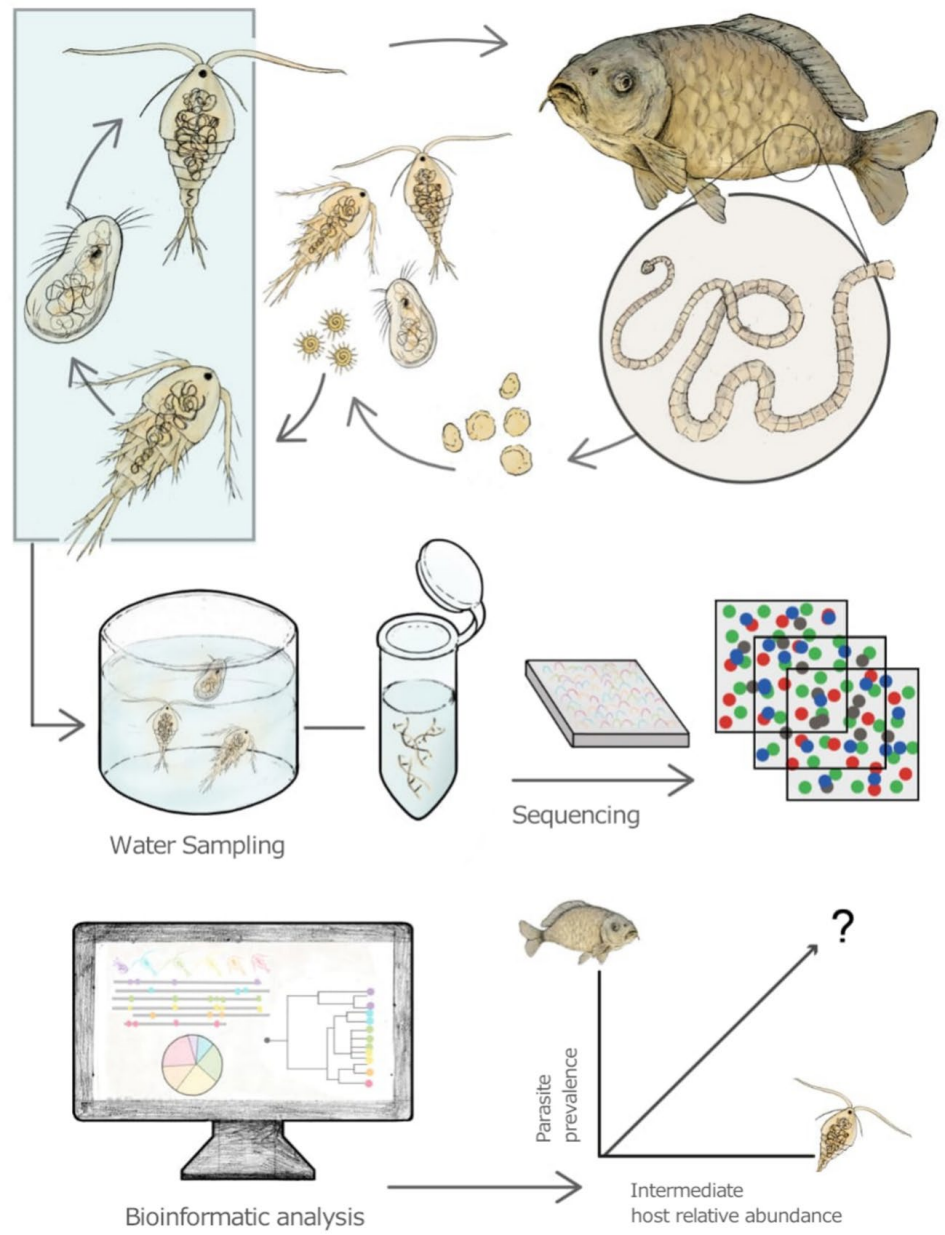
Conceptual workflow of eDNA applications for the detection of *Schyzocotyle acheilognathi* in copepod intermediate and fish final hosts. Environmental DNA from freshwater ecosystems can be used to determine intermediate and final hosts from sediment and water column samples. In general, we propose identifying the copepods within the planktonic community and associated with infected fish populations. Upon collection of plankton samples, eDNA can be extracted to amplify specific barcoding regions. These amplicons can be further sequenced massively. Sequencing data can be processed using available computational pipelines that utilize public or customized reference databases of barcodes. Upon applying statistical analysis, the contribution to transmission of particular intermediate hosts could be assessed by analyzing the association between parasite prevalence in definitive hosts and the abundance of particular copepod species whose abundance is strongly correlated to AFT infection

In Fig. 5, we present a conceptual framework for further analysis of copepod communities cohabiting with *S. acheilognathi*-infected fish. This general framework can be useful as a starting point to identify copepod taxa that potentially host this parasite. However, further validation would be necessary: once a copepod candidate has been identified, it could be experimentally tested. For example, Hansen et al. (2007) evaluated the impact of food availability and moderate cold shock on bonytail chub fish, *Gila elegans*, parasitized with AFT and observed that infested fish reached lower sizes and became more vulnerable to death. Although these authors did not evaluate the likelihood of fish becoming infected due to the consumption of infested copepods, this kind of bioassay could be useful to test it.

Another important aspect that warrants further exploration is the differential susceptibility observed among certain taxonomically related species. For example, Pinacho-Pinacho et al. (2015) reported the incidence of AFT in Mesoamerican fish of the family Profundulidae, specifically in *Profundulus guatemalensis*, *Tlaloc hildebrandi*, *T. labialis*, and *T. portillorum*. These authors found that while this host family generally has low susceptibility to *S. acheilognathi*, *T. hildebrandi* is highly susceptible (Velázquez-Velázquez et al. 2015). Although these conclusions may be influenced by the differences in the sample size among studies (*e.g*., in *T. hildebrandi*, more than 1000 specimens were reviewed, and temporal variation was analyzed, while in the rest of the species, only 58 specimens were screened) (Velázquez-Velázquez et al. 2011), it is also likely that *T. hildebrandi* possesses intrinsic factors that make it susceptible to this cestode. If this assertion is true, then it could represent an opportunity to investigate the genomic and other intrinsic host features underlying susceptibility. Furthermore, because *T. hildebrandi* has a highly restricted distribution, it could be useful to associate the local environmental characteristics with parasite load to determine the ecological factors that may influence this interaction.

It is also important to determine whether AFT has intermediate hosts other than copepods. In this respect, only one experimental study has indicated that ostracods can be intermediate hosts (Marcogliese and Esch 1989), but some authors maintain that larger crustaceans can also serve as intermediate hosts (Boomker et al. 1980; Paperna 1996; Kuchta et al. 2018; Skelton 2024).

### Additional opportunities for the implementation of genomic approaches for the study of AFT

#### Genomic and functional perspectives

Just as there is a clear need to explore ecological, biogeographical, and demographic dimensions of AFT, there is also an immense opportunity to investigate its cellular and molecular foundations (Kokova and Mayboroda 2019; Attenborough et al. 2024). Holistic perspectives integrating molecular mechanisms into the study of organismal evolution have expanded rapidly since the beginning of the twenty-first century. The advent of omics tools—such as genomics, transcriptomics, and proteomics—has revolutionized our understanding of many pathogens and their interactions with hosts (Wang et al. 2019, 2021; Doyle 2022; Rubio-Godoy et al. 2025). However, the adoption of these tools among parasitologists has been uneven, and fish helminths like AFT remain poorly represented in genomic repositories such as WormBase ParaSite (Howe et al. 2017).

Given the parasite’s ecological and economic relevance (Kuchta et al. 2018), implementing omic approaches could reveal how AFT adapts to diverse environments and hosts. These methods may uncover mechanisms of immune evasion, genomic erosion due to parasitism, or transcriptional regulation across developmental stages—questions essential for understanding its evolutionary success and informing management strategies (Wang et al. 2019, 2021; Buzy et al. 2021; Airs et al. 2022).

#### Symbiotic microbiota and host interactions

Recent interest in parasite-associated microbiota provides another valuable perspective for exploring the adaptability of AFT. Early studies (Fu et al. 2022) suggested that the Asian fish tapeworm harbors a distinct microbiota influencing host gut communities. However, this idea has been re-evaluated by Casanova-Hernández (n.d.) *in press*, who analyzed AFT specimens from various fish hosts—*C. carpio*, *P. bimaculatus*, *T. hildebrandi*, and *Vieja hartwegi*—collected under natural conditions. Their findings show no evidence of a consistent, intrinsic microbial community in the parasite, nor of dysbiosis in parasitized hosts, reinforcing the idea that this parasite lacks an intrinsic bacterial community (Hammer et al. 2017, 2019; Casanova-Hernández n.d. *in press*).

The bacterial assemblages detected in AFT were highly variable among hosts, suggesting that bacteria are externally acquired through stochastic processes rather than maintained symbiotically. While certain taxa such as *Cetobacterium* appeared recurrently—consistent with Fu et al. (2022)—their presence likely reflects transient associations derived from the host environment (Bledsoe et al. 2016; Ramírez et al. 2018; Bi et al. 2021). This remarkable microbial plasticity mirrors that seen in other generalist or free-living helminths (Jorge et al. 2020; Scott 2023; Trejo-Meléndez and Contreras-Garduño 2024) and correlates with host traits and ecological conditions (Amillano-Cisneros et al. 2022; Sparagon et al. 2022; Araujo et al. 2025).

This is important from an applied but also from a fundamental perspective since the number of symbiotic bacteria identified for helminths is scarce (Vaughan et al. 2012; Bouchery et al. 2013; Reynolds et al. 2015; Ashour and Othman 2020; Jorge et al. 2022a, b). Although the existence of an accompanying microbiota might confer some advantage in the establishment of some parasites, as symbiotic associations are a fast way to gain novel functions, as occurs with some ectoparasites, in the case of AFT, it is probable that these beneficial interactions do not exist except for adult parasites (René-Martellet et al. 2017; Ponnusamy et al. 2018; Ben-Yosef et al. 2020; Casanova-Hernández et al. n.d. *in press*). However, it is also possible that AFT selects specific microbes at each stage of its life history to confer different adaptive traits required in specific stages of its development while not maintaining core taxa that accompany it along its life cycle, as occurs with other species. For example, a study conducted by Jorge et al. (2020) demonstrated that the trematode *Coitocaecum parvum* possesses a phylogenetically diverse microbiota distinct from its host or external factors that varies according to the developmental stage but that also includes a core microbiota present in all life stages (Jorge et al. 2022a, b). The survey of the bacterial communities present at each life cycle stage of the parasite could provide clues on the roles played by tightly associated microbiota in invasiveness, virulence, and novel host-parasite interactions (Dheilly et al. 2015, 2019). For example, Martinson et al. (2020) analyzed the role of the microbiota of the nematode *Howardula aoronymphium* infesting *Drosophila* flies. They found that the reduction of the bacterial communities and the elimination of the endosymbiont *Candidatus Symbiopectobacterium* using antibiotic therapy provoked an infection decrease. Another example involving *Zeacumantus subcarinatus* snails infected with metacercariae of the trematode *Philophthalmus attenuatus* and exposed to antibiotic mixtures resulted in negative effects on the parasite (Jorge et al. 2022b).

Another opportunity area is the development of novel non-invasive molecular tests for the detection of AFT. Current methods for detecting its presence require destructive sampling of fish, which must be dissected for the direct observation of the parasite. In some cases, such as when surveying fish species at risk of extinction, the excessive collection of specimens can cause disturbances for the stability of the surveyed populations. In these scenarios, the use of non-invasive, DNA-based methods is encouraging, particularly considering their proven utility in monitoring parasite communities (Avramenko et al. 2018; Gogarten et al. 2019) or the parabiome, which refers to the composition of helminth and protozoan parasite communities in a host (Thorn et al. 2023). These kinds of studies have centered on nematodes. For example, Avramenko et al. (2015) proposed the metabarcoding of nematodes using the ITS-2 rDNA region for larval identification. However, the application of this approach in fish parasites remains much less common compared to its extensive use in terrestrial hosts such as calves (Avramenko et al. 2017; De Seram et al. 2022), roe deer (Beaumelle et al. 2022), horses (Poissant et al. 2021), bisons (Avramenko et al. 2018), or primate species (see Gogarten et al. 2019). These studies can help estimate parasite diversity and identify co-occurring parasites, but none of these approaches have been used for the AFT.

## Conclusion

Exploratory analyses indicate that temperature, precipitation, and elevation are key variables shaping the distribution of AFT. These findings provide a foundation for more comprehensive ecological niche models aimed at predicting potential expansion under future environmental and climatic scenarios. Extending such analyses to regional and global scales could help identify environmental thresholds for establishment and highlight host species or geographic areas at greatest risk of AFT infection/invasion. Integrating ecological records and published data, we found no evidence that any particular fish taxonomic group or size class is uniquely susceptible to infection.

Despite its broad host range and global distribution, AFT exhibits low genetic diversity, yet this pattern may underlie a form of evolutionary resilience. Whether such reduced variability reflects an adaptive strategy promoting ecological plasticity or phenotypic stability remains unresolved and warrants further investigation through integrative genomic approaches.

In conclusion, future research should prioritize the identification and characterization of intermediate host populations through combined ecological, molecular, and experimental approaches. The integration of high-throughput omics and environmental DNA tools could further elucidate mechanisms of host adaptation and enable non-destructive detection of infections in natural systems. Such efforts will advance our understanding of the ecological dynamics, transmission potential, and evolutionary responses of AFT across diverse freshwater environments.

## Data Availability

Data used in this study is fully accessible in supplementary tables.
